# Fibroblast–immune crosstalk in oral squamous cell carcinoma: from tumor promotion to immune evasion

**DOI:** 10.3389/fimmu.2025.1703277

**Published:** 2026-01-09

**Authors:** Xiao Liu, Dandan Wang, Xue Cheng, Yining Ma, Siya Li, Jinhai Deng, Chunyan Qiao

**Affiliations:** 1 Hospital of Stomatology, Jilin University, Changchun, China; 2 Jilin Provincial Key Laboratory of Tooth Development and Bone Remodeling, Changchun, China; 3 Chinese Academy of Sciences (CAS) Blue Bay Cloud Technology (Guangdong) Co., Ltd., Guangzhou, China; 4 Guangzhou Baiyunshan Pharmaceutical Holding Co., Ltd. Baiyunshan Pharmaceutical General Factory, Guangdong Province Key Laboratory for Core Technology of Chemical Raw Materials and Pharmaceutical Formulations, Guangzhou, China

**Keywords:** cancer-associated fibroblasts, extracellular matrix, immune crosstalk, immune suppression, metabolic reprogramming, oral squamous cell carcinoma, tumor microenvironment

## Abstract

Oral squamous cell carcinoma (OSCC) is a highly invasive malignancy marked by poor prognosis and therapeutic resistance. Within its tumor microenvironment (TME), cancer-associated fibroblasts (CAFs) and immune cells form a dynamic network that drives tumor progression. CAFs reshape the extracellular matrix, rewire tumor metabolism, and modulate immune surveillance by recruiting regulatory T cells and promoting macrophage polarization. Recent studies have highlighted CAF heterogeneity, identifying functionally distinct subtypes with differential impacts on prognosis and treatment responsiveness. CAF-derived secretomes, including cytokines, chemokines, and exosomal miRNAs, shape tumor–immune dynamics and mediate resistance to cisplatin and anti-angiogenic therapies. Crosstalk between CAFs and immune cells, particularly via the IL-6–STAT3–PD-L1 and CXCL12–CXCR4 axes, promotes immune evasion and dampens responses to checkpoint blockade. This review summarizes the phenotypic heterogeneity, metabolic functions, and secretory profiles of CAFs in OSCC, with particular emphasis on their crosstalk with immune components, highlighting the potential of CAFs-targets to enhance immunotherapy responsiveness.

## 1 Introduction

Oral squamous cell carcinoma (OSCC), a prevalent and aggressive malignancy within the head and neck region, continues to present substantial clinical challenges despite advances in conventional treatment modalities (1, 2). Patients with OSCC frequently experience poor outcomes characterized by high rates of local recurrence, lymph node metastasis, and persistently low five-year survival (3), which underscore the urgent need to identify novel molecular targets and implement innovative therapeutic paradigms. The tumor microenvironment (TME), a dynamic and heterogeneous niche composed of cellular and acellular components, critically influences carcinogenesis, tumor progression, and responsiveness to therapeutic interventions (4–6). Rather than functioning as passive structural support, the TME integrates stromal elements, vasculature, lymphatic networks, and extracellular matrix components (7), wherein fibroblasts, endothelial cells, and diverse immune populations orchestrate complex signaling programs that regulate tumor behavior and drive therapeutic resistance (8, 9).

Within the TME, cancer-associated fibroblasts (CAFs) constitute the predominant population of stromal cells, exerting broad and dynamic control over cancer progression (10, 11). By extensively modifying the extracellular matrix and releasing a diverse spectrum of signaling molecules and extracellular vesicles, CAFs orchestrate the behavior of neighboring malignant and non-malignant cells. These concerted actions foster a supportive niche conducive to tumor expansion, tissue invasion, neovascularization, immune suppression, and the epithelial–mesenchymal transition (EMT) process (10, 12, 13). This review summarizes the multifactorial roles of CAFs in the TME of OSCC, offering insight into therapeutic interventions that target CAFs as a novel modality for OSCC treatment.

## 2 Overview of CAFs

### 2.1 Phenotypic markers of CAFs

CAFs are broadly recognized as a dominant non-malignant component embedded within the tumor stroma. Histologically, these cells are distinguished in clinical specimens by their absence of markers associated with epithelial, endothelial, and hematopoietic lineages. Additionally, CAFs do not exhibit the somatic genomic alterations characteristic of tumor cells and display an elongated spindle-like morphology under microscopy (14, 15). Although it is now accepted that CAFs may arise from a diverse range of progenitors, including tissue-resident fibroblasts, bone marrow-derived mesenchymal stem cells, pericytes, and adipocytes—the precise ontogeny of these cells remains incompletely delineated, likely owing to the complex interplay of local microenvironmental cues (14–16). A variety of molecular markers have been utilized to identify CAFs, among which α-smooth muscle actin (α-SMA), fibroblast activation protein (FAP), fibroblast-specific protein 1 (FSP1), vimentin, and platelet-derived growth factor receptors alpha and beta (PDGFR-α/β) are commonly cited (17). It is important to note that CAF marker expression profiles exhibit tumor-type specificity (18). In head and neck cancers, CAFs are typically characterized by expression of α-SMA, FAP, PDGFR-α/β, FSP1, NG2, and PDPN. Specifically in OSCC, α-SMA, FAP, and vimentin serve as the principal identifiers (12). CAF activation is driven by several well-characterized molecular and cellular mechanisms. These include activation of the transforming growth factor-beta signaling axis, direct physical interaction between carcinoma cells and fibroblasts, exposure to inflammatory cytokines, dynamic remodeling of the extracellular matrix, oxidative stress and metabolic reprogramming leading to the accumulation of reactive oxygen species, and genotoxic insults resulting from chemotherapy or ionizing radiation (14).

### 2.2 CAF subtypes and functional roles

Emerging evidence highlights the pronounced phenotypic and functional diversity of CAFs across tumor types (18). While quiescent CAFs exhibit features akin to normal fibroblasts, their activated counterparts acquire a pro-tumorigenic phenotype. CAFs have been stratified into at least three distinct populations: myofibroblastic CAFs, inflammatory CAFs (iCAFs), and antigen-presenting CAFs (19). myCAFs are typically enriched in peritumoral regions, display elevated α-SMA expression, and do not produce IL-6. In contrast, iCAFs are spatially more distant from malignant clusters, lack α-SMA, and robustly express IL-6. A third subset, apCAFs, has thus far been characterized exclusively in murine models and remains undetected in human specimens (15, 17, 20, 21). Although progress has been made, the comprehensive delineation of CAF heterogeneity within OSCC remains largely unexplored. These diverse fibroblast populations are increasingly recognized as pivotal modulators of the TME, with the potential to reshape the biological and immunological landscape of the tumor (7). Functionally, CAFs contribute to a spectrum of pro-tumorigenic activities, including restructuring of the extracellular matrix, reprogramming of cellular metabolism, and dynamic modulation of immune surveillance. Furthermore, they facilitate tumor cell proliferation, drive angiogenic processes, enable EMT, and enhance migratory and invasive capacities. In parallel, CAFs support the maintenance of cancer stem cell-like traits and underlie mechanisms of therapeutic resistance, underscoring their significance as integral players in OSCC progression and treatment response (12, 16). Recent studies have begun to uncover OSCC-specific CAF subsets with distinct functional roles (22). In particular, CD68^+^ CAFs have been linked to improved patient outcomes, in contrast to their CD68^−^ counterparts, which are more strongly associated with immunosuppressive phenotypes and unfavorable prognosis (23). Mechanistically, CD68^+^ CAFs appear to enhance anti-tumor immune responses by promoting the secretion of CCL17 and CCL22, which facilitate regulatory T cell recruitment in a potentially regulatory feedback context (24). Conversely, CD68^−^ CAFs are enriched in immunosuppressive cytokines and are correlated with increased infiltration of myeloid-derived suppressor cells (MDSCs) and M2-polarized macrophages (25, 26). Similarly, α-SMA^+^ CAFs, enriched in peritumoral regions, are characterized by high contractile activity and the secretion of pro-tumorigenic mediators such as TGF-β1, IL-6, and CXCL12, which drive EMT, angiogenesis, and M2 macrophage recruitment (27–29). These CAFs activate signaling pathways including RhoA/ROCK, JAK/STAT3, and MAPK, fostering tumor progression (30, 31). These findings underscore the relevance of CAF subset profiling for prognostication and therapeutic stratification in OSCC (**Supplementary Table S1**).

## 3 CAFs regulate tumor environment in OSCC

### 3.1 CAF-mediated extracellular matrix remodeling and metabolic reprogramming

In the tumor microenvironment of OSCC, CAFs represent a dominant stromal component and play a central role in reconfiguring the ECM to facilitate malignant progression (32, 33). Through the activation of focal adhesion kinase (FAK) signaling, CAFs can enhance the expression of lysyl oxidase (LOX) (34). This cascade results in pronounced ECM contraction, a reduction in collagen pore dimensions, and an increase in matrix stiffness—structural changes that collectively support OSCC cell proliferation, motility, invasion, and the induction of EMT (35, 36). Moreover, CAFs influence cytoskeletal architecture and promote the nuclear translocation of Yes-associated protein (YAP) through the RhoA–ROCK axis. Once localized in the nucleus, YAP reprograms fibroblast chromatin states and contributes to matrix stiffening, further augmenting OSCC cell invasiveness (37–39). By persistently remodeling the ECM, CAFs establish a permissive microenvironmental scaffold that guides tumor cell dissemination (40). Notably, epidermal growth factor (EGF) has been shown to synergize with CAF-derived cues to potentiate ECM restructuring and enhance the invasive behavior of head and neck squamous cell carcinoma (HNSCC) cells (41, 42).

CAFs exert profound control over tumor metabolic plasticity, influencing key pathways of glucose, amino acid, and lipid metabolism within the tumor microenvironment (43–45). CAFs enhance aerobic glycolysis via the upregulation of 6-phosphofructo-2-kinase/fructose-2,6-biphosphatase 3 (PFKFB3), facilitating lactate production. This glycolytic output, driven by the lncRNA H19/miR-675-5p/PFKFB3 axis, sustains OSCC cell proliferation and contributes to an immunosuppressive metabolic milieu (46, 47). Suppressing PFKFB3-mediated glycolytic flux leads to activation of the PGC-1α axis, ultimately impairing the angiogenic support function of CAFs (48). By contrast, upregulation of TRAP1 in CAFs inhibits mitochondrial oxidative phosphorylation (OXPHOS), exerting a suppressive effect on the progression of OSCC (49). Moreover, CAF-specific integrin beta-2 (ITGB2) overexpression activates the PI3K/AKT/mTOR pathway, increasing lactate secretion (50, 51). Once exported via MCT4 and taken up by OSCC cells through MCT1, lactate is converted to NADH, fueling mitochondrial oxidative phosphorylation and promoting tumor proliferation and EMT (52, 53). In OSCC, CAFs reprogram glutamine metabolism by expressing glutamine synthetase, thereby generating glutamine *de novo* (53). This glutamine is transferred to adjacent tumor cells, where it is converted to glutamate by glutaminase and subsequently fuels the TCA cycle, supporting rapid biosynthesis and redox homeostasis (46, 54). This metabolic support is particularly critical in nutrient-deprived microenvironments, allowing OSCC cells to sustain anabolic growth and resist oxidative stress. CAFs also reshape lipid metabolic networks by upregulating fatty acid synthase (FASN), leading to increased fatty acid secretion (55, 56).

### 3.2 Secretory functions of CAFs in OSCC progression

In OSCC, CAFs orchestrate diverse oncogenic processes through an extensive secretome. They enhance the expression of oncogenic mediators including activin A, fibronectin type III domain-containing protein 1 (FNDC1), SERPINE1, and stanniocalcin 2 (STC2) (57). Notably, a marked reduction of exosomal miR-34a-5p is observed in CAFs, and its reintroduction impairs tumorigenic potential *in vivo* by downregulating AXL, thereby suppressing both proliferative and metastatic capabilities (58, 59). In OSCC, CAF-derived exosomes enriched in miR-382-5p contribute to the invasiveness of malignant cells (60). Moreover, exosomal miR-192/215 released under hypoxic conditions by HNSCC cells suppresses caveolin-1 (CAV1) expression in fibroblasts, thereby triggering TGF-β pathway activation and reprogramming the hypoxic tumor niche (61). Integrated transcriptomic and proteomic analyses have identified a long noncoding RNA, termed lnc-CAF, that promotes the transition from normal fibroblasts to CAFs via IL-33–dependent pathways; silencing this RNA dampens tumor expansion and reduces levels of α-SMA^+^ CAFs and Ki-67 expression (62, 63). Under hypoxic conditions, CAFs secrete serglycin (SRGN), which facilitates the nuclear accumulation of β-catenin in head and neck squamous cell carcinoma (HNSCC), thereby activating Wnt/β-catenin signaling and promoting cellular stemness and drug resistance (64). In parallel, CAF-released hepatocyte growth factor (HGF) activates c-Met in tumor cells, leading to metabolic rewiring through modulation of glycolytic enzymes such as hexokinase-II and phosphofructokinase, alongside increased MCT1 activity. Conversely, basic fibroblast growth factor (bFGF) produced by tumor cells stimulates MAPK signaling in CAFs, enhancing both their proliferation and subsequent HGF secretion (65, 66).

The remodeling of the ECM byc-derived matrix metalloproteinases (MMPs) contributes to a permissive niche for invasion (67). During dynamic OSCC–CAF interactions, these fibroblasts produce a broad spectrum of proinflammatory and angiogenic cytokines—including TGF-β, VEGF, TNF-α, HGF, IL-1α/β, IL-6, and IL-33—that collectively support invasive behavior and metastatic dissemination (68, 69). Moreover, autocrine IL-1β activates TRAF6, amplifying paracrine TNFα signaling and potentiating the invasive phenotype of squamous carcinoma cells (70, 71). Epiregulin (EREG), enriched in CAFs, drives fibroblast activation and EMT via JAK2–STAT3 and IL-6 pathways; three-dimensional *in vitro* systems validate its contribution to OSCC invasiveness (72). IL-6 secreted by CAFs amplifies osteopontin expression via activation of the NF-κB signaling pathway, thereby enhancing the proliferative, invasive, and migratory behavior of head and neck cancer cells (73). Concurrently, tumor-derived IL-1β transforms resident fibroblasts into CAFs that subsequently secrete CXCL1, which stimulates the CXCR signaling cascade to potentiate metastatic dissemination (74). Angiogenic processes in OSCC are further accelerated through CAF-derived TNFα and SDF1/CXCL12, which act through the CXCR4 signaling axis (7, 75), along with elevated secretion of IL-6 and VEGF (76). Following CXCL12 binding, endothelial CXCR4 is activated, triggering PI3K/AKT and MAPK/ERK cascades that promote endothelial proliferation, migration, and survival (77, 78). Concurrently, CAF-secreted MMPs, particularly MMP-2 and MMP-9, degrade basement membrane components and remodel the extracellular matrix to create pro-angiogenic niches (79, 80). These signals converge on the activation of angiogenesis-associated transcription factors such as HIF-1α and NF-κB, which transcriptionally upregulate pro-angiogenic genes, thereby sustaining capillary sprouting and vascular maturation (81). CAFs also support angiogenesis by releasing proteolytic enzymes and remodeling extracellular matrix components, thereby facilitating endothelial migration and capillary formation. Additionally, extracellular vesicles from CAFs and the activation of PGC-1α/PFKFB3 metabolic signaling further reinforce glycolytic flux and angiogenic capacity (48, 82). Direct cell–cell interaction with OSCC cells induces NOTCH3 expression in CAFs, which further contributes to the angiogenic remodeling of the TME (83).

## 4 Immune cell-CAF crosstalk in OSCC

### 4.1 Modulation of the tumor microenvironment by CAF-secreted molecules

**Figure 1:**
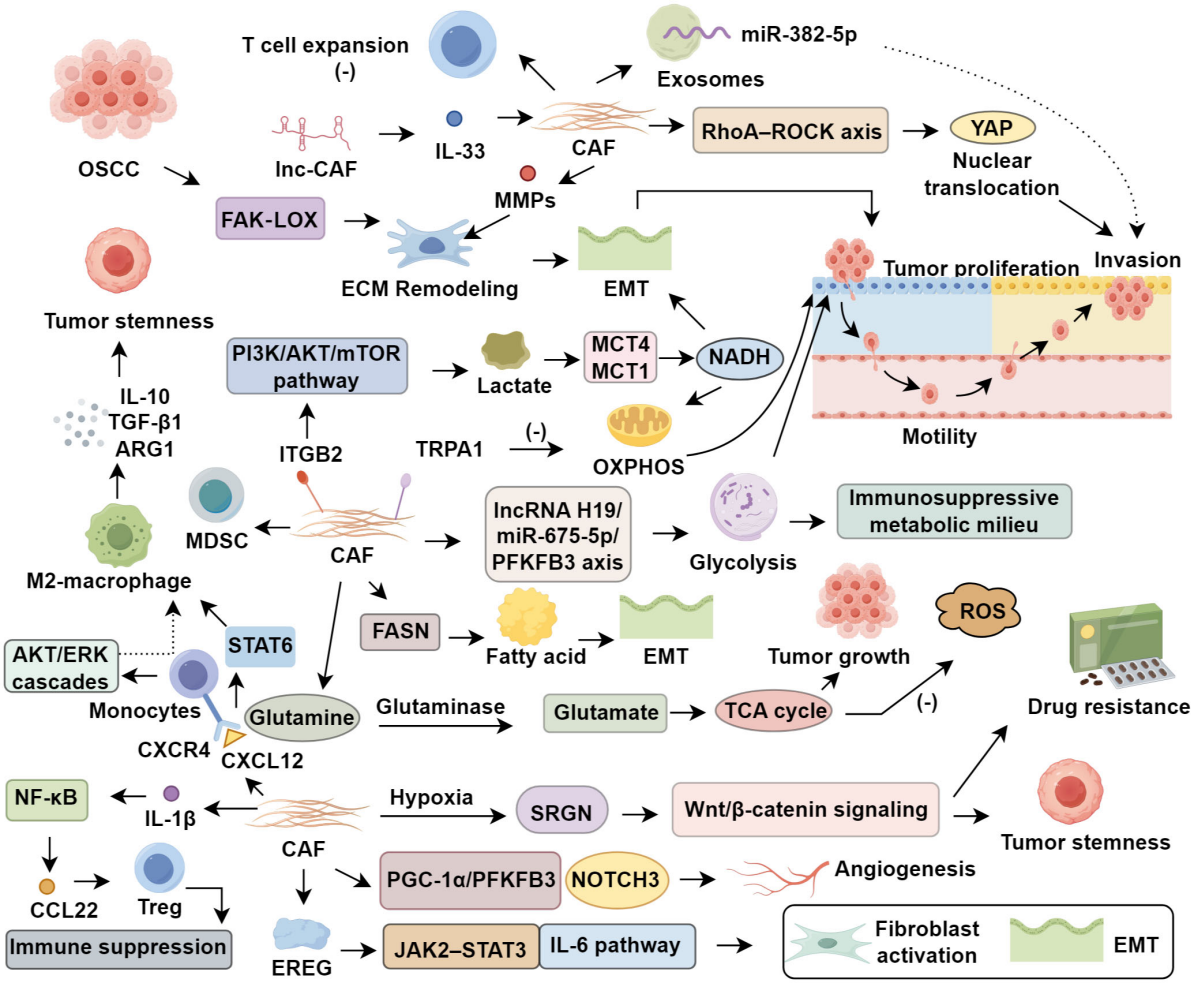
Roles of cancer-associated fibroblasts in oral squamous cell carcinoma.

As integral constituents of the TME, CAFs exert profound influence on the oncogenic niche through both ECM remodeling and secretion of bioactive factors. Among these, IL-6 serves a dual function by not only promoting neoplastic cell proliferation but also sustaining CAF activation, thereby reinforcing a mutualistic feedback loop between stromal and malignant cells (84). This dynamic interplay is initiated by TGF-β, which induces fibroblast activation; in response, CAFs secrete a repertoire of effector molecules, including TGF-β1, HGF, fibroblast growth factors (FGFs), stromal cell–derived factor 1 (SDF-1), CCL2, and IL-6, which collectively reprogram the TME and influence cellular migration and invasive behavior (85, 86). Additionally, CAFs contribute to ECM restructuring through the enzymatic activity of MMPs and the RhoA–ROCK pathway, thereby generating physical conduits that facilitate tumor cell motility, invasiveness, and resistance to therapeutic agents (80, 87). Moreover, endothelial cells exposed to both TNF-α and TGF-β undergo EMT and subsequently produce cytokines that amplify EMT in OSCC, further accelerating tumor advancement (88, 89). CAF-secreted IL-1 has also been shown to reshape the TME, thereby promoting OSCC invasiveness (90). CAF-secreted IL-6 can activate STAT3 signaling in tumor and immune cells, which in turn upregulates PD-L1 expression and impairs CD8^+^ T cell–mediated cytotoxicity (84, 91). This IL-6–STAT3–PD-L1 axis has emerged as a critical mechanism by which CAFs establish an immunosuppressive niche and promote resistance to immune checkpoint inhibitors (92–94). As such, targeting this signaling cascade may offer a promising strategy to overcome immunotherapy resistance in OSCC and other solid tumors (**Figure 1**).

### 4.2 Immune-suppressive roles of CAFs

Within the tumor microenvironment, CAFs actively contribute to immunosuppressive remodeling through multiple regulatory circuits. One such mechanism involves the secretion of Wnt family member 2 (WNT2), which suppresses dendritic cell–driven immune activation via modulation of the SOCS3–JAK2–STAT3 signaling cascade (95). In addition, CAF-derived IL-1β initiates transcriptional upregulation of the chemokine CCL22 in tumor cells through NF-κB activation, thereby facilitating regulatory T cell (Treg) recruitment and reinforcing local immune suppression (96, 97). A distinct CAF subset exhibiting diminished CD68 expression has been linked to elevated infiltration of Tregs and unfavorable clinical outcomes in OSCC cohorts (24). Moreover, CAF-secreted CXCL12 engages CXCR4 receptors on monocytes, activating AKT and ERK signaling cascades that promote polarization toward an M2-like macrophage phenotype (98). These M2 macrophages secrete immunosuppressive cytokines (IL-10, TGF-β1) and arginase 1 (ARG1), fostering a pro-tumorigenic microenvironment and enhancing OSCC cell stemness and proliferative capacity (75). The CXCL12/CXCR4 axis operates as a central conduit for CAF-driven immune suppression: binding of CXCL12 to CXCR4 on myeloid progenitors promotes STAT6-mediated transcription of M2-polarization genes (IL10, MRC1), which in turn accelerates TAM-mediated angiogenesis and immune evasion in OSCC (99, 100). Inhibiting this axis has shown promise in preclinical models by restoring T cell infiltration and enhancing checkpoint blockade efficacy (101). Through the production of pro-inflammatory mediators, CAFs also facilitate the recruitment of tumor-associated macrophages (TAMs), promoting immune evasion (25). In murine models of early-stage OSCC, tumor-derived IL-1α amplifies the immunosuppressive activities of both CAFs and TAMs. During metastatic progression, CAFs further potentiate the accumulation of MDSCs, exacerbating immune tolerance within the TME (102). CAFs impede T cell expansion by expressing co-inhibitory ligands and secreting immunosuppressive cytokines. This dual action not only drives apoptosis in effector T cells but also enhances Treg proliferation, thereby cementing an immunosuppressive ecosystem in OSCC (82, 103, 104).

## 5 CAFs in drug resistance and sensitivity of OSCC

Autophagy plays a dual role in modulating chemotherapy responses, wherein its activation can protect tumor cells from cisplatin-induced apoptosis by enabling cellular recycling and stress adaptation. CAFs promote chemoresistance in OSCC through dual mechanisms. Firstly, they enhance the expression of autophagy-related proteins LC3 and Beclin1, which diminishes the efficacy of cisplatin (105). Secondly, midkine (MK)-enriched CAFs confer resistance by upregulating the long non-coding RNA ANRIL in tumor cells. ANRIL knockdown disrupts drug transporters MRP1 and ABCC2, inhibits proliferation, induces apoptosis, and ultimately sensitizes cells to cisplatin. This MK-mediated resistance operates through a caspase-3 and Bcl-2–dependent pathway, as evidenced by the fact that exogenous MK can restore the resistant phenotype (106). Furthermore, CAFs facilitate both neovascularization and resistance to bevacizumab by releasing extracellular vesicles that present surface-bound VEGF ligands (107, 108). Extracellular vesicles derived from cancer stem cells in OSCC can induce the transformation of fibroblasts into CAFs, which subsequently reinforce tumorigenic potential and mediate resistance to cisplatin (109). Exosomes secreted by CAFs containing miR-196a enhance proliferative capacity and cisplatin tolerance in head and neck cancer cells by directly targeting CDKN1B and ING5 (110). Moreover, CAFs critically contribute to ECM restructuring, modulation of metabolic pathways, immune evasion, and resistance to anticancer therapies (111). A functionally distinct CAF subset expressing NOTCH3 has been linked to angiogenic processes that drive OSCC progression (112). Given these precedents, therapeutic interventions directed against NOTCH3-positive CAFs in OSCC hold the potential to attenuate tumor-associated angiogenesis and impede disease advancement. Additionally, NADPH oxidase 4 (NOX4) plays a pivotal role in facilitating myofibroblast-to-CAF differentiation. Pharmacological blockade of NOX4 not only prevents the acquisition of CAF-like features but also curtails OSCC tumor development, highlighting the therapeutic potential of targeting CAF plasticity (113, 114).

## 6 Conclusion

Cancer-associated fibroblasts represent a central orchestrator of OSCC progression, modulating nearly every aspect of the tumor microenvironment, including matrix architecture, metabolic plasticity, immune suppression, and drug resistance. The heterogeneity of CAF subsets, ranging from tumor-promoting to tumor-suppressive phenotypes, underscores the necessity for precise molecular stratification prior to therapeutic intervention. Promising approaches such as targeting SPARC-mediated nanoparticle uptake, HSF1 and TGF-βRII signaling, or CAF-derived exosomal miRNAs, offer innovative avenues to disrupt tumor–stroma crosstalk. However, indiscriminate CAF depletion may impair immune surveillance or promote tumor aggressiveness.

To advance CAF-directed therapy in OSCC, several critical knowledge gaps must be addressed. First, a comprehensive mapping of CAF subtypes specific to OSCC is urgently needed to elucidate the cellular diversity and context-dependent functions of these stromal cells. Second, robust and clinically applicable biomarkers capable of stratifying patients based on CAF activity or abundance are lacking but essential for guiding personalized treatment decisions. Third, the integration of CAF molecular signatures into existing immunotherapy stratification frameworks may enhance the predictive power and therapeutic responsiveness of immune checkpoint blockade or combination regimens. Future efforts must prioritize selective reprogramming of pro-tumor CAFs while preserving or enhancing anti-tumor subsets, thereby enabling tailored stromal therapies that complement conventional and immune-based regimens in OSCC.
